# Beyond effectiveness: orienting youth mental health research towards population impact

**DOI:** 10.3389/frcha.2026.1952905

**Published:** 2026-10-07

**Authors:** M. E. Loades, J. R. Weisz, R. S. Beidas

**Affiliations:** 1 Department of Psychology, University of Bath, Bath, United Kingdom; 2 University of Bath Mental Health Research Group (MHRG), Bath, United Kingdom; 3 Department of Psychology, Harvard University, Cambridge, MA, United States; 4 Department of Medical Social Sciences, Feinberg School of Medicine, Northwestern University, Chicago, IL, United States

**Keywords:** implementation science, mental health interventions, population impact, scale-up and sustainment, youth mental health

## Abstract

Youth mental health problems are a major and growing global challenge. Although effective interventions exist for many common mental health problems, population-level improvements have been limited. This paradox highlights that intervention effectiveness alone is likely insufficient to generate meaningful public health impact. Interventions must also reach young people, be adopted by services and practitioners, be delivered with quality, and be sustained over time. Drawing on implementation science, we argue that youth mental health research should ask not only “Does this intervention work?” but also “Can this intervention generate population-level impact?”. We illustrate how impact considerations can be embedded throughout the intervention lifecycle. First, we discuss designing for reach and adoption, using single-session interventions as an example of scalable approaches aligned with real-world patterns of service use. Second, we highlight the importance of evaluating effectiveness under routine conditions, drawing on a trial of a modular treatment in outpatient child and adolescent clinics as an example of generating externally valid evidence. Third, we emphasise the need to study implementation and sustainment, using examples from national mental health programmes and implementation research initiatives. To reduce the burden of youth mental health problems, implementation, reach, and sustainment must be treated as core objectives if our work is to have population-level impact.

## Introduction

The growing burden of youth mental health problems is a global concern (1). Numerous interventions for common youth mental health problems have been found effective in clinical trials (2, 3) but these interventions do not reach the vast majority of youths who need help (4, 5). This is a critical challenge for the field. Demonstrating that an intervention is effective is necessary, but it is not sufficient to generate population-level impact. For the purposes of this perspective, we use population-level impact to refer to the extent to which an intervention improves mental health outcomes across a population by being adopted into and delivered with fidelity and quality within routine practice settings, reaching substantial numbers in those settings, and being sustained over time, based on Dodge et al. (6). Implementation science frameworks such as RE-AIM (7) have, for years, stressed that public health impact depends on multiple interacting dimensions, including effectiveness, reach, adoption, implementation, and maintenance (8). Yet, youth mental health intervention research continues to prioritise efficacy evidence while often treating implementation as a downstream consideration—that is, someone else's job.

If our goal is to reduce the burden of youth mental health problems at scale, we need to move beyond asking “Does this intervention work?” and also ask “Can this intervention generate population-level impact?”. This requires implementation considerations to be embedded throughout the intervention lifecycle, and success should be judged not only by whether interventions improve outcomes for individual participants, but by whether they achieve meaningful population-level improvements for many through broad reach, equitable access, widespread adoption, high-quality implementation, and long-term sustainment (6). Herein, we illustrate how this shift in focus can be operationalised through examples from youth mental health research and practice. In this article, we use the term youth mental health problems to refer primarily to emotional and behavioral difficulties and disorders among children and young people. We do not seek to encompass all issues affecting youth wellbeing or child protection, although these may intersect with mental health and influence implementation.

## Designing for reach and adoption

If interventions are to achieve population-level impact, they must be designed for reach and adoption as well as effectiveness. Decisions about how we provide mental health support to youth often prioritise “gold standard” interventions based on efficacy and effectiveness evidence, most commonly multi-session therapies evaluated in controlled trials (9). The treatment protocols in 443 randomised controlled trials (RCTs) specified a mean of 16.54 sessions (SD 14.06) (3). Yet, many youth who access help in real world settings do not complete the full course of treatment available to them (10) and attend for very few sessions (11, 12) with a substantial minority only attending once (13). Designing interventions with these usage patterns in mind could increase reach and adoption and thereby boost population impact. When designing interventions, we should not only ask whether an intervention can work under ideal circumstances, but whether it is likely to be used by young people and providers in the first place, and if so, whether the intervention can work when used as it is most likely to be used in everyday practice (14).

One example of a scalable format for intervention delivery that aligns with real world use patterns and is designed for adoption and reach is the single-session intervention approach (SSI). SSIs are intentionally designed to stand-alone (rather than to presume repeated use or multisession attendance) and have demonstrated effectiveness in reducing mental health symptoms and improving functioning (15), including when offered to youth as self-help (16). Self-help, digitally delivered SSIs are inherently scalable and have been shown to be feasible and acceptable in a range of real-world settings where youth spend time, as in schools in the USA (17, 18), England (19), Kenya (20), Ukraine (21) and Lebanon (22), and with refugee youths displaced from their own country by war (23). Furthermore, they can be offered via widespread public health messaging online and in community settings (24–28), and as well as in physical healthcare settings (29). Key implementation considerations addressed at the formative stages have included (1) how to ensure that youth are empowered to consent for themselves (30, 31) and (2) where and how to let youth know about these interventions, including via social media (32, 33). By contrast, treatment completion for scalable, multisession, self-directed, open-access internet-delivered cognitive behavioural therapy (iCBT) for anxiety has been low in real-world settings (34), potentially because the multisession format does not align with actual usage patterns: in one large Australia-wide programme, less than a third of registered participants completed three or more of the available 10 sessions, while 21.7% did not complete the first session (34).

## Evaluating effectiveness in real-world conditions

Population impact depends on effectiveness in the contexts where interventions are actually delivered (6, 35), and efficacy evidence derived from highly controlled conditions may not accurately reflect outcomes achieved in routine care. An example is a RCT conducted across 10 outpatient clinical service organisations in Massachusetts and Hawaii (36). The intervention was delivered by the existing workforce of 84 clinicians, reflecting typical service conditions. The study compared a modular treatment approach with usual care and standard evidence-based treatments for children aged 7–13 years referred with anxiety, depression, or conduct problems. The modular approach outperformed both comparison conditions on multiple clinical outcomes, including weekly symptom measures and diagnostic outcomes, and participants showed faster rates of improvement. Sessions delivered using the modular approach also contained substantially higher levels of evidence-based content than sessions provided under usual care (83% compared with 8%). Interestingly, evidence-based content was most evident in the standard condition (93%), which was associated with weaker clinical outcomes than the modular condition. This suggests the possibility that extreme fidelity to treatment manual content may not work as well in actual clinical care settings as a more balanced approach that emphasizes evidence-based elements while leaving room for unscripted clinician-youth interactions. In this and many other ways, embedding trials within clinical care can generate interesting hypotheses about ways practitioners may make evidence-based practice both ecologically sensitive and effective.

Alongside effectiveness outcomes, cost and resource requirements for delivery are also important implementation considerations. Even interventions that are relatively simple to deliver may require investment in staff time, training, infrastructure, supervision or ongoing technical support, which may influence whether they are adopted and sustained at scale.

## Studying implementation and sustainment at scale

Even when interventions are shown to be effective and achieve widespread adoption, relatively little research is directed towards whether effects are sustained at scale; this limits our understanding of their population-level impact. To bridge this gap, robust outcome evaluation must be embedded within large-scale implementation efforts, including national rollouts. In England, Mental Health Support Teams (MHSTs) that provide early help for common mental health problems in the form of brief, low intensity therapies, delivered by trained graduate mental health workers, have been nationally implemented in schools. Routine outcome measurement, using standardised and psychometrically validated tools, is mandated within service specifications to individual level measurement-based care and enable broader service evaluation. However, because data collection is managed at the service level, there are significant inconsistencies in how data are recorded, making it difficult to aggregate data across services (Woods et al., forthcoming). In contrast, NHS Talking Therapies for adults (formerly IAPT) use a centralised system for outcome data, which has enabled large-scale research and service evaluation. Analyses of these routinely collected data have identified variation in access, engagement, and outcomes across services and population groups, informing quality improvement efforts and providing evidence about how evidence-based interventions perform in routine practice at scale (37, 38). A youth specific example of a programme of implementation research work which specifically aimed to evaluate implementation strategies and pathways to the adoption of an evidence based strategy is a programme of work on firearms storage in paediatric health settings in the USA (39).

Hybrid effectiveness–implementation studies could narrow the gap between research and population-level impact by simultaneously generating evidence about both whether interventions work and how they can be delivered effectively in practice (40), and should become the norm rather than the exception in child and adolescent mental health research. Lorente-Catala (41) demonstrate how hybrid studies can simultaneously answer effectiveness and implementation questions in the context of depression in primary care settings in Spain. There are also ongoing randomised controlled trials of brief, scalable treatments for adolescent depression in England including an RCT of an imagery based intervention delivered in schools (42) to inform a future hybrid effectiveness-implementation trial, and a hybrid type 1 effectiveness-implementation RCT of self-help single session interventions as a source of on demand and barrier free support (Loades et al, forthcoming). Both trials seek to explore factors impacting treatment delivery and to report treatment costs alongside effectiveness outcomes.

Importantly, the implementation of youth mental health interventions extends beyond individual services or settings. Youth interact with multiple systems, including schools, primary and specialist healthcare, community organisations and social care, and implementation is also shaped by wider policy, commissioning and funding contexts. Sustaining effective intervention delivery may therefore require coordination across the ecosystem rather than focusing solely on implementation within a single setting (43). This broader systems co-ordination is reflected in approaches such as the US Systems of Care approach (44) and the UK THRIVE Framework, which seeks to organise mental health support around the needs of children, young people and families across different levels of need and service contexts (45).

## Discussion

The key take-away is that population impact = real-world effectiveness + reach + adoption + implementation + sustainment. The considerations we have described map onto established implementation outcomes, including adoption, acceptability, appropriateness, costs, feasibility, fidelity, sustainability and penetration (46). To improve mental health at population level, it is necessary, but not sufficient to know what works and how well it works under ideal conditions; we must also understand how, when, where, and for whom interventions work in practice. Rather than viewing implementation as the final stage of a research pathway, we should treat it as a core scientific concern throughout the intervention lifecycle throughout intervention development, evaluation, and scale-up (see Table 1).

**Table 1:** Implementation priorities across the lifecycle of youth mental health interventions.

Research Stage	Primary implementation priorities	Key implementation considerations	Example questions
Intervention Development	Acceptability, appropriateness, feasibility, equity, usability	Engage young people, families, practitioners, and service throughout intervention design. Understand contextual barriers and facilitators. Design for accessibility, inclusivity, affordability, and compatibility with routine practice. Consider scalability from the outset.	Who is the intervention intended to reach, and who might be excluded? Does it address priorities identified by young people and services? Can it be delivered within existing resource constraints? What adaptations may be needed for different populations or settings?
Evaluation	Reach, engagement, fidelity, adoption, cost, effectiveness across contexts	Measure implementation outcomes alongside clinical outcomes. Examine who engages with the intervention, how it is delivered in practice, and what resources are required. Assess variation in implementation and outcomes across settings and populations.	Who accesses and completes the intervention? How acceptable and feasible is delivery in routine settings? What factors influence uptake and engagement? Does effectiveness differ across contexts, service settings, or demographic groups? What are the costs of implementation?
Scale-up and sustainment	Penetration, sustainability, adaptation, workforce capacity, system integration	Develop strategies for wider adoption and long-term delivery within the ecosystem of provision. Build organisational readiness and workforce capability. Establish sustainable funding and monitoring systems. Support appropriate adaptation while maintaining core intervention functions.	What conditions are needed for successful scale-up? How can the intervention be integrated into existing systems and pathways? What training, infrastructure, and funding are required? How will fidelity, adaptation, and impact be monitored over time? Does the intervention continue to reach and benefit young people at scale?

Implementation in youth mental health also involves considerations that are specific to this population. These include developmental differences in needs and decision-making, and also balancing the involvement of parents and caregivers with increasing youth autonomy and independence. Furthermore, legal issues around age of consent, confidentiality and information sharing, as well as the roles of schools and other youth-serving systems, including child welfare and juvenile justice. These factors can create additional implementation challenges and require coordination across health, education, social care and community settings.

Realising the vision of a deployment-focused model of treatment development (43, 47–49) will require shifts in research culture, funding priorities, training, and evaluation practices (50). An enduring emphasis on effectiveness over a broader range of implementation outcomes, limited implementation science capacity, and structural constraints within youth services all risk slowing progress. Addressing these challenges will require stronger partnerships between researchers, practitioners, policymakers, and young people, alongside greater investment in implementation-focused research. Only through such changes can we ensure that effective interventions translate into meaningful population-level improvements in young people's lives.

## Data Availability

The original contributions presented in the study are included in the article/Supplementary Material, further inquiries can be directed to the corresponding author.
